# Validation and Extension of a Fluid–Structure Interaction Model of the Healthy Aortic Valve

**DOI:** 10.1007/s13239-018-00391-1

**Published:** 2018-11-07

**Authors:** Anna Maria Tango, Jacob Salmonsmith, Andrea Ducci, Gaetano Burriesci

**Affiliations:** 1 UCL Mechanical Engineering, Cardiovascular Engineering Laboratory, University College London, Torrington Place, London, WC1E 7JE UK; 2 Bioengineering Group, Ri.MED Foundation, Via Bandiera 11, 90133 Palermo, Italy

**Keywords:** Fluid–structure-interaction (FSI), Valsalva sinus, Heart valve dynamics, Haemodynamics

## Abstract

**Purpose:**

The understanding of the optimum function of the healthy aortic valve is essential in interpreting the effect of pathologies in the region, and in devising effective treatments to restore the physiological functions. Still, there is no consensus on the operating mechanism that regulates the valve opening and closing dynamics. The aim of this study is to develop a numerical model that can support a better comprehension of the valve function and serve as a reference to identify the changes produced by specific pathologies and treatments.

**Methods:**

A numerical model was developed and adapted to accurately replicate the conditions of a previous *in vitro* investigation into aortic valve dynamics, performed by means of particle image velocimetry (PIV). The resulting velocity fields of the two analyses were qualitatively and quantitatively compared to validate the numerical model. In order to simulate more physiological operating conditions, this was then modified to overcome the main limitations of the experimental setup, such as the presence of a supporting stent and the non-physiological properties of the fluid and vessels.

**Results:**

The velocity fields of the initial model resulted in good agreement with those obtained from the PIV, with similar flow structures and about 90% of the computed velocities after valve opening within the standard deviation of the equivalent velocity measurements of the *in vitro* model. Once the experimental limitations were removed from the model, the valve opening dynamics changed substantially, with the leaflets opening into the sinuses to a much greater extent, enlarging the effective orifice area by 11%, and reducing greatly the vortical structures previously observed in proximity of the Valsalva sinuses wall.

**Conclusions:**

The study suggests a new operating mechanism for the healthy aortic valve leaflets considerably different from what reported in the literature to date and largely more efficient in terms of hydrodynamic performance. This work also confirms the crucial role that numerical approaches, complemented with experimental findings, can play in overcoming some of the limitations inherent in experimental techniques, supporting the full understanding of complex physiological phenomena.

**Electronic supplementary material:**

The online version of this article (doi:10.1007/s13239-018-00391-1) contains supplementary material, which is available to authorized users.

## Introduction

The comprehensive understanding of the optimum haemodynamic environment that regulates the operating mechanisms of the healthy aortic valve is essential in enabling a correct interpretation of diseased conditions and their implications, and to devise effective therapies that restore or mimic the crucial physiological functions. It is therefore understandable that a substantial amount of literature has been produced on the topic.

Whilst there is consensus that the haemodynamics established within the aortic root plays a key role in the proper valve function^4^,^38^,^40^,^48^ and optimum flow to the coronary arteries, there is no agreement on the specific mechanisms involved.^3^,^36^,^49^ Although the Valsalva sinuses are commonly indicated to promote fluid recirculations, which in turn act upon the leaflets, some investigations report that these vortices form within the sinuses in early systole,^15^,^33^,^52^ while others claim that these structures only occur during late systole.^16^,^35^,^43^ The number and locations of these vortices are also disputed, with contrasting research indicating multiple vortices within each sinus,^15^ a single vortex fully contained within each sinus^43^ or a vortex only partially within the sinus.^16^,^35^ Consequently, the basic understanding of the native aortic valve’s operating process is still fragmentary.

The need for a better insight into the establishment of optimum fluid dynamics in the aortic valve region has now become a timely and critical issue, due to the significant correlation with clinical complications recently reported with surgical and transcatheter bioprosthetic replacements, which can be associated with the non-physiological flow environment that they produce.^11^,^12^,^18^ Unfortunately, *in vivo* studies based on techniques such as magnetic resonance imaging (MRI) and ultrasound present practical limitations in measuring the velocity field, due to their reduced spatial and temporal resolution^39^; and *in vitro* studies, although capable of capturing the main flow features downstream of the valve, only allow the measurements in limited regions, normally outside of the valve structure.^2^,^12^,^13^,^31^,^54^

In this scenario, the opportunity to adopt numerical models to comprehend the complex dynamics of the valve is apparent. In fact, *in silico* simulations can offer a comprehensive representation of the valvular structures and flow dynamics across the valve at different spatial and temporal scales. This requires the development of high resolution fluid–structure interaction (FSI) models, which allow the description of both the mechanical behavior of the tissue components and the fluid dynamics throughout the cardiac cycle, which play a significant role towards the achievement of a more extensive understanding of the valve function.^53^ Nevertheless, due to the complexity of the valve dynamics, this numerical approach can easily lead to erroneous findings, and the correct tuning of the involved parameters is crucial to attain trustworthy representations of the studied phenomena. This has been clearly acknowledged by the International Organization for Standardization (ISO) working group,^50^ which strongly endorses the combination of advanced experimental and computational studies to obtain reliable results from complex numerical simulations.^53^

This synergistic approach was adopted in the present study, with the aim to understand the optimum flow dynamics that should be expected within a healthy and ideal aortic root, in physiologically normal operating conditions. In particular, a preliminary numerical model was designed to replicate a previous *in vitro* study performed by our group^47^ that, with all inherent limitations intrinsic in the experimental simulation, had attempted to model healthy native physiological conditions. The flow in that study was determined and analysed at specific instants of the systolic cycle by means of 2D particle image velocimetry (PIV). The experimental velocity fields obtained from this *in vitro* analysis are here used to achieve a quantitative and qualitative validation of the numerical model at the available instants, whilst a global verification of the acceptability of the computational simulation was confirmed *via* comparison of the effective orifice area (EOA), a parameter recommended by the ISO to quantify the systolic valve performance.

Once the numerical framework used to construct the initial model was validated by the results from the previous *in vitro* study, the simulation was altered to better represent an idealized healthy aortic valve. Some of the major limitations affecting the benchtop investigation, such as the presence of the bioprosthetic valve stent, the rigid material used for the mock aortic root and the use of a test fluid denser than blood, could be corrected in a modified computational model, based on the same framework as the validated model.

## Methodology

### Numerical Methodology

Numerical analyses were performed on an Intel Core i7 3.4 GHz workstation using the explicit finite element software LS-DYNA Release 9.2 (LSTC, Livermore, CA, USA). This software specializes in non-linear transient dynamic problems, suitable for investigating complex phenomena involving large deformations, advanced material models and fluid–structure coupling.^23^ The package has been previously used for the analysis of heart valve fluid dynamics,^6^,^9^,^25^,^34^,^36^,^43^ and is recommended by the ISO working group as a commercial FSI software for the assessment of potential thrombus formation in heart valve implants.^50^

Simulations were performed by coupling a Lagrangian model of the aortic root and leaflets with an Eulerian fluid domain *via* a hybrid Arbitray-Lagrangian-Eulerian (ALE) algorithm.^29^ The solid structures were immersed in the fluid control volume, where each domain was modeled independently without the need of a conforming mesh at the fluid–structure boundary.^32^ An “operator split” technique was used to solve the fluid domain^23^ and the ALE algorithm coupled the two domains, performing a remeshing of the Lagrangian elements only.^7^,^32^,^51^ The fluid motion equations were solved by splitting the time integration cycle into two steps: a Lagrangian time step, and a “remap” or “advection” step, where an advection term is applied to remap the fluid domain to its original configuration.^1^ Although the fluid grid may distort in the Lagrangian phase, the solution must be referred back to the undistorted initial frame during the advection phase.^9^

### Validation of Preliminary Numerical Model

#### Geometry and Meshing

A preliminary computational model was created to replicate the *in vitro* configuration previously used to perform a fluid dynamic investigation of the aortic valve by means of PIV analysis.^47^ This included a rigid silicone root of geometry based on the description of the healthy human anatomy provided by Swanson and Clark,^44^ in reference to an annulus equal to 25 mm, corresponding to an average healthy adult.^46^ The aortic root cross section was approximated by an epitrochoid function assuming identical dimensions for the three Valsalva sinuses, as suggested by Reul *et al*.^37^ The root hosted a 29 mm Labcor (Labcor Laboratórios Ltd., Belo Horizonte, Brazil) stented porcine bioprosthesis. This surgical valve size was selected because its leaflets were of similar size to those of a native aortic valve with a 25 mm annulus.^47^ In the experimental study, a groove was made in the silicone root to embed the stent and the sewing ring, thus reducing flow perturbations induced by the presence of these components. However, in order to maintain the correct position of the leaflets with respect to the aortic root, small portions of the frame remained exposed at the base of the Valsalva sinuses. In addition, the aortic chamber was rigid rather than compliant and, since it resulted unachievable to create a test fluid with the same viscosity and density as human blood whilst maintaining the required refractive index matching between the solution and the silicone root, higher fluid density than blood had to be accepted for the blood substitute solution.

**Figure 1:**
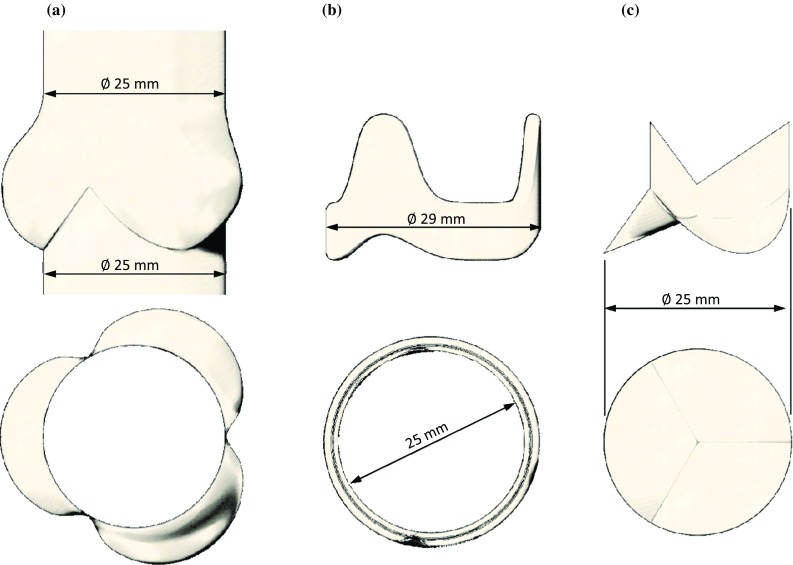
Sketch of the aortic root (a), the valve stent (b) and the leaflets (c) geometries used in the numerical model.

The preliminary numerical model replicated the inner surface of the idealized aortic root (without including the groove needed in the physical model to host the stent and sewing ring) and the external surfaces of the stent geometry, as measured from the physical model (Figs. 1a and 1b). Compenetration between the two components was allowed, mimicking the embedding of the stent into the groove of the mock root. As the shape of the leaflets is very complex to replicate,^53^ and the intent of the study was to analyse a generalized configuration representative of ideal native conditions, the leaflet geometry adopted for the numerical model was based on the description of the idealized healthy human aortic valve provided by Thubrikar^45^ (Fig. 1c) for an annulus diameter equal to 25 mm.

The aortic root, the stent and the valve leaflets were discretized into 9960, 6852 and 6564 4-noded Belytschko-Tsay shell elements,^23^ respectively. Shell elements were preferred for the modeling of thin-walled structures, due to their computational efficiency compared to solid elements.^9^ In fact, the ability to include several integration points through their thickness allows more accurate modeling of the bending of non-linear material models, with no significant increase in the computational time.^34^ The aortic leaflets and wall thicknesses were considered to be uniformly distributed with a value of 0.5 mm^26^,^35^,^41^ and 3 mm,^43^ respectively. The attachment nodes of the leaflets were shared with the elements at the base of the Valsalva sinuses and commissural lines of the root.

**Figure 2:**
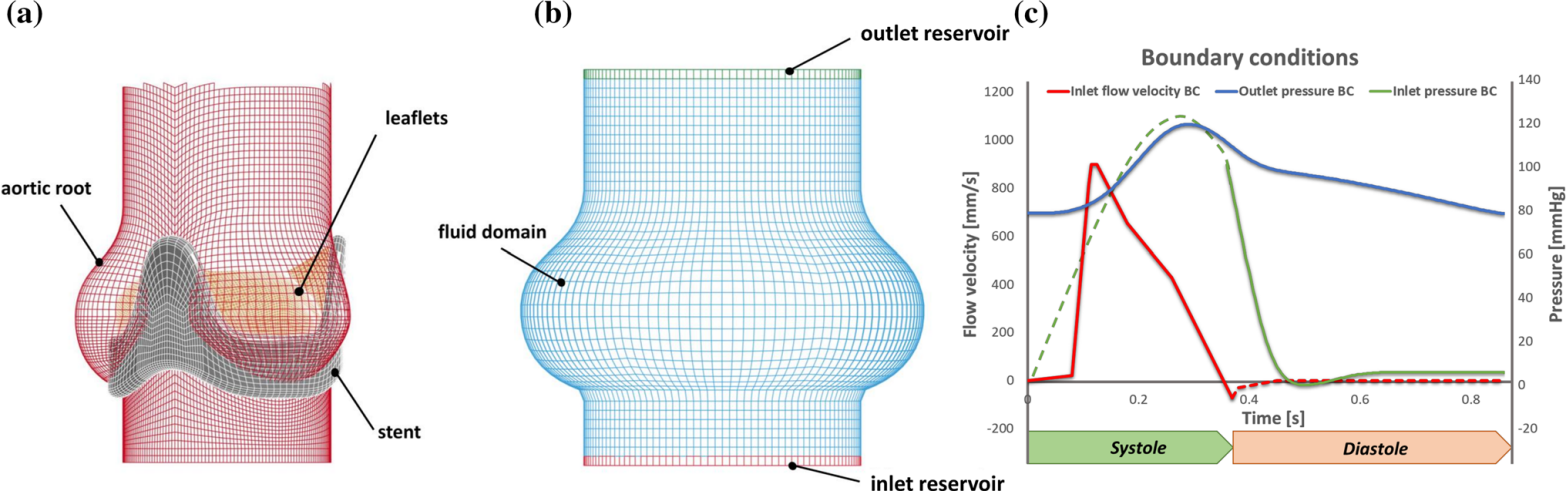
(a) Mesh of the structural components in the validation model, including the aortic root, the stent, and the leaflets; (b) mesh of the fluid domain, with the inlet and outlet reservoirs; and (c) pressure and velocity waveforms applied as boundary conditions to the surface of the fluid domain in contact with the inlet and outlet reservoir.

Since Thubrikar’s description of the aortic leaflets provides information to generate a fully closed valve configuration, whilst the bioprosthetic porcine valve used for the *in vitro* validation was characterized by a semi-open shape when at rest in saline solution, a pre-expansion procedure was undertaken. To achieve this, the leaflets were initially modeled as linear elastic, with a Young’s modulus of 1 MPa and a Poisson’s ration of 0.45, and then expanded by applying a uniformly distributed opening pressure of 5 mmHg. The resulting configuration, which resulted similar to that observed for the prosthetic porcine leaflets used in the *in vitro* experiment, was then adopted as the initial unloaded shape, by rezeroing the stresses and strains in the model. The resulting model of the structure is represented in Fig. 2a.

The fluid was discretised into a structured mesh of 113,520 8-noded hexahedral Eulerian elements with a characteristic dimension of 1 mm, which satisfied a convergence analysis (for further details refer to Appendix A1 in the Electronic Supplementary Material). The choice of hexahedral elements was based on their superior performance in the FSI algorithm compared to tetrahedral elements, which typically leads to reduced accuracy and numerical instability during the remap phase.^29^ Two reservoirs were created at the level of the inlet and outlet (Fig. 2b), made of elements capable of supplying and absorbing fluid.^9^ These were used to impose the fluids’ boundary conditions, which were applied as a combination of physiological flow velocity and pressure differences, as described below. Finally, the control volume and reservoirs’ mesh grids were discretised respecting the Valsalva sinuses symmetry, in order to guarantee a proper setting of the boundary conditions on the fluid domains, as recommended by Luraghi *et al*.^30^

#### Materials Modeling: Aortic Tissues and Blood Properties

The aortic root and the valve stent were modeled as perfectly rigid in the preliminary numerical model, to better replicate the negligible compliance of the mock root and the high stiffness of the stent in the experimental study.^47^ Leaflets were approximated as homogeneous isotropic membranes,^24^ neglecting the influence of their complex multilayer histological microarchitecture.^5^ Their non-linear constitutive behavior was modeled as hyperelastic and incompressible, adopting an Ogden’s formulation. This is suitable for describing the mechanical properties of complex materials such as rubbers, polymers, and biological tissues.^55^

The Ogden model expresses the strain energy (*W*) by principal stretches (*λ*_*α*_), *α *= 1, 2, 3^21^:

1 $$W = \mathop \sum \limits_{p = 1}^{N} \frac{{\mu_{p} }}{{\alpha_{p} }} \left( {\lambda_{1}^{{\alpha_{p} }} + \lambda_{2}^{{\alpha_{p} }} + \lambda_{3}^{{\alpha_{p} }} - 3} \right)$$

where $\mu , \alpha$and *N* are material characteristic constants. The material constants used in the analysis were taken from a previous study,^8^ and fitted using a four parameters equation with *μ*_1_ = 7.6 × 10^−6^ MPa; *μ*_2_ = 5.7 × 10^−4^ MPa; *α*_1_ = *α*_2_ = 26.26. A density of 1100 kg/m^3^ was selected, as this is typical for biological soft tissues.^19^

In the validation study, the fluid was modeled as Newtonian, isothermal and incompressible, with a dynamic viscosity of 4 × 10^−3^ Pa s and density equal to 1294 kg/m^3.^^47^ These match the physical properties of the test fluid used for the *in vitro* experiment selected for validation.^47^

#### Boundary Conditions

An aortic pressure waveform was prescribed at the outlet cross section throughout the cardiac cycle, oscillating between 80 mmHg (diastolic) and 120 mmHg (systolic). For the inlet boundary conditions, the velocity flow waveform measured from the *in vitro* study used for validation^47^ was applied during systole. This enforced healthy physiological conditions at rest characterized by a cardiac output of 4 l/min, a heart rate of 70 bpm with 35% of systolic time, and a mean aortic pressure of 100 mmHg. Due to the proximity of the root to the ventricular chamber, which is substantially shorter than its entry length, the velocity was uniformly distributed over the inlet cross section. In order to best simulate the closing dynamics, which involves the closing leakage produced by the reversal of the transvalvular pressure difference, during diastole the velocity profile was replaced by the application of a ventricular pressure waveform, establishing the pressure drop measured during the same phase in the *in vitro* test.^47^ This approach is reported to be appropriate to capture the physiological valve opening-closure mechanics.^27^

The boundary conditions applied to the fluid are summarized in Fig. 2c.

Three consecutive cycles were run, discarding the results from the first cycle and using the other two to confirm that cyclic stability in the predicted flow parameters was achieved.

#### Validation

Results were analysed using Ls-PrePost 4.3 and Paraview 5.4.1 post-processing software. The data from the last of the three simulated cycles of the numerical model were used to extract the velocity contour maps and the corresponding velocity vectors.

The velocity fields of the sagittal aortic root cross section from the numerical study, matching the cross section analysed in the *in vitro* study, were compared with the experimental measurements at the 4 different instants of the cardiac cycle analysed with PIV in the *in vitro* experiment. The time instants considered corresponded to the following flowrate conditions: maximum increasing flowrate (Instant ‘A’), peak flowrate (‘B’), maximum decreasing flowrate (‘C’) and end of systole zero flowrate (‘D’).

The numerical velocity fields at each instant were qualitatively compared and validated against those obtained from the experiment, focusing on the flow distribution, direction and magnitude in the sinuses and central jet, as well as on the presence, development, size and (where applicable) direction of vortices and stagnant regions.

A quantitative validation was carried out by comparing the evolution, with respect to time within each cycle, of the velocity across the full field. The experimental data were plotted together with their standard deviation to take into account the cyclic variation of the velocity fields.

For further quantitative validation, the downstream velocities across the root diameter aligned with one of the commissures, at the height of the STJ, were extracted from both studies and compared (for the *in vitro* analysis, the standard deviation was included).

The EOA was used as a quantitative parameter describing the global hydrodynamic performance across the whole simulated cycle for both the experimental and numerical analyses. Based on the international standard ISO 5840 recommendation, this is estimated as:

2 $${\text{EOA}} = \frac{{q_{{v{\text{RMS}}}} }}{{51.6\sqrt {\frac{\Delta p}{\rho }} }},$$

where $q_{{v{\text{RMS}}}}$ is the root mean square forward flow during the positive differential pressure period, expressed in ml/s; $\Delta p$ is the mean pressure difference measured during the positive differential pressure period, expressed in mmHg; and *ρ* is the density of the test fluid, expressed in g/cm^3^^20^ Hence, this parameter takes into account both the flowrate and the transvalvular pressure difference during the entire systolic phase.

### Physiological Healthy Model

**Figure 3:**
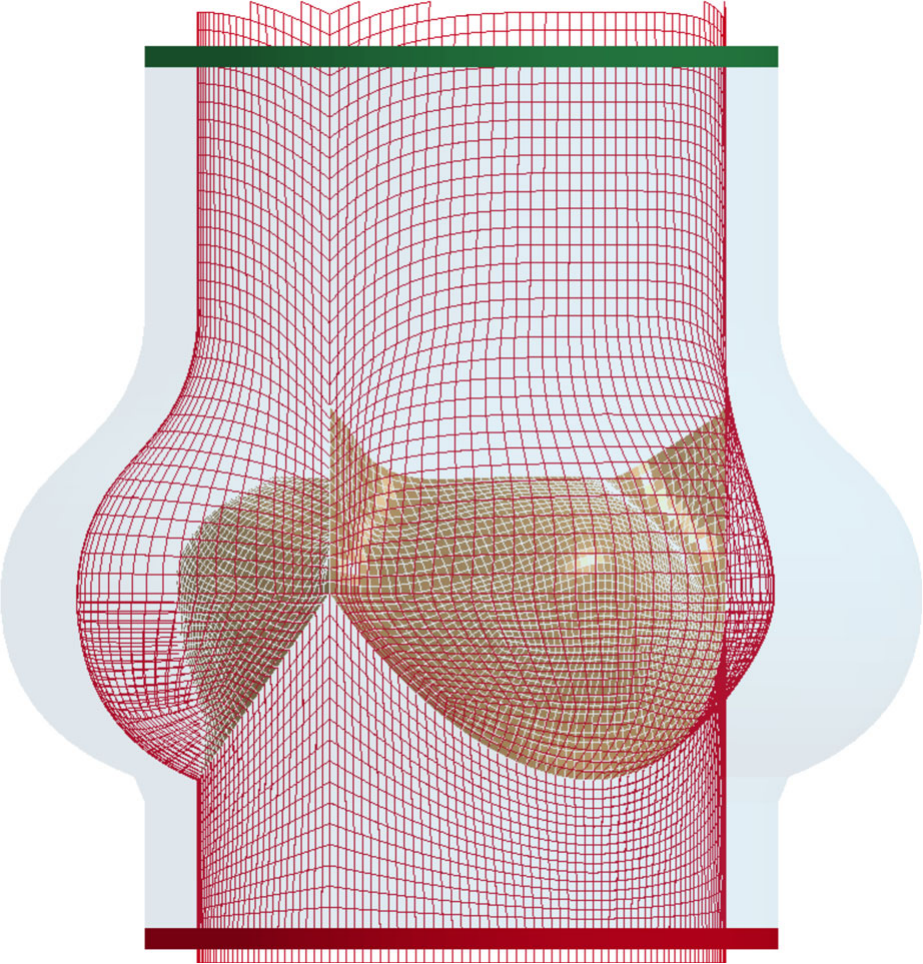
Mesh of the structural components in the physiological model, including the aortic root and the leaflets. The fluid domain, with the inlet and outlet reservoirs is unchanged, as represented by colored regions.

Once the preliminary model had been validated by comparison with the *in vitro* experimental data, modifications were made to provide a more accurate description of the healthy physiological condition. This was achieved by: (a) removing the presence of the stent and sewing ring; (b) including the compliance of the root walls, and (c) adjusting the physical properties of the fluid, to match that of healthy human blood. In particular, the aortic wall’s material was modeled as linearly elastic, with a Young’s modulus of 3.25 MPa and a Poisson’s ration of 0.45. These were estimated to match the vessel compliance value of a normal healthy aorta, as recommended in the international standard ISO 5840 (*C* = 0,32%/mmHg).^17^ No change was introduced in the constitutive model of the leaflets compared with the model implemented for validation. The fluid was maintained Newtonian, as this is considered acceptable by the ISO standards for the levels of shear rates and vessel diameters involved in the study.^50^ Its density was reduced to 1060 kg/m^3^, corresponding to the standard value for healthy human blood. The geometries and mesh of the root, leaflets and fluid domain were left unaltered, (see Fig. 3) as well as the boundary conditions prescribed to the fluid reservoirs. In order to avoid a significant change in the shape of the pressurized aorta, a uniformly distributed pressure equal to 80 mmHg, directed inwards, was applied to all elements of the root wall above the leaflets’ attachment.

## Results

### Validation of Preliminary Model

**Figure 4:**
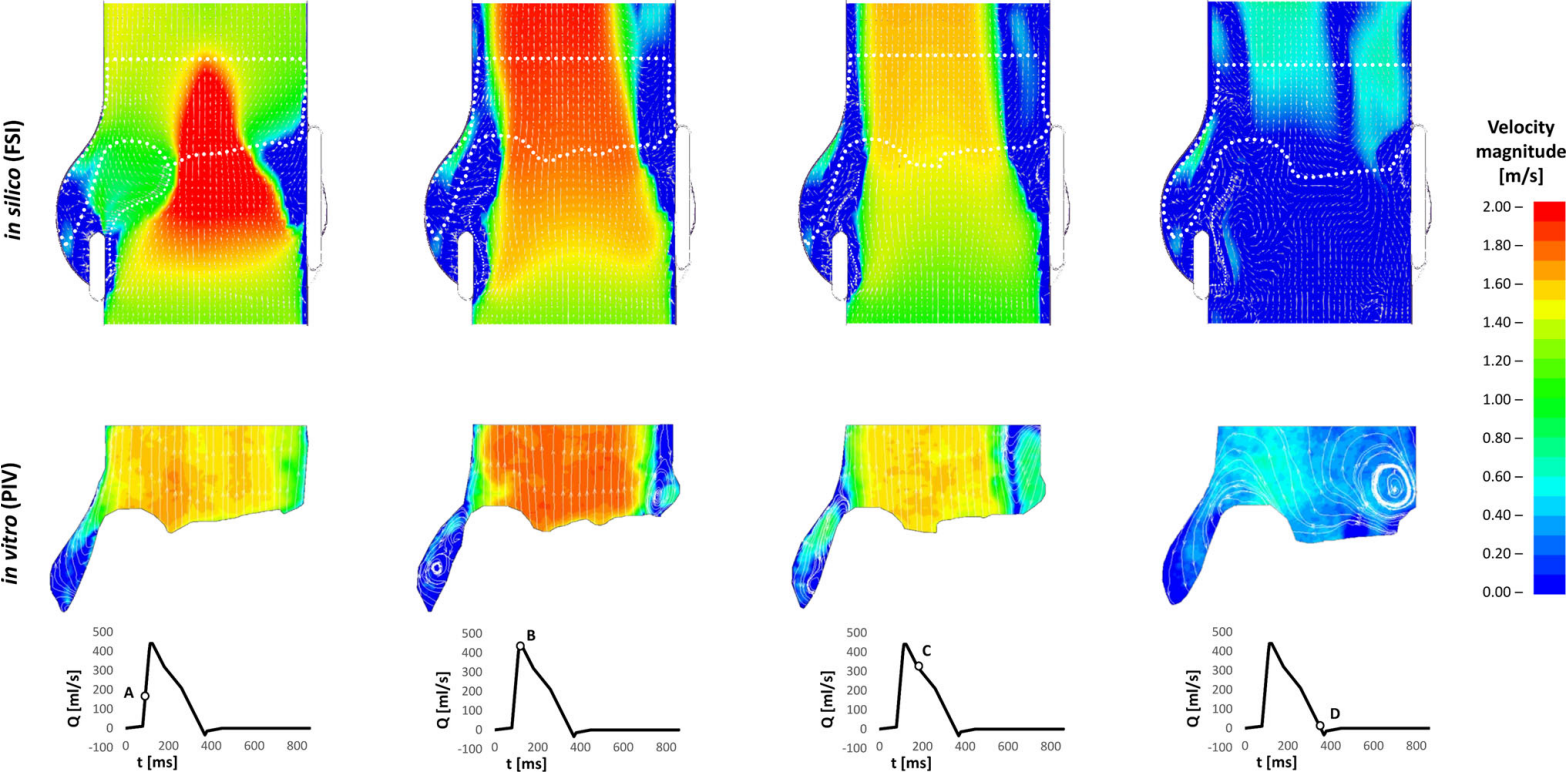
Comparison between *in silico* FSI and *in vitro* PIV of the flow velocity map and vectors fields at instants ‘A’, ‘B’, ‘C’ and ‘D’ of the cardiac cycle.

The velocity fields of the sagittal aortic root cross section for the preliminary numerical model and the PIV analyses are represented in Fig. 4. Due to optical obstruction from the pulse duplicator and the shadow produced by the leaflets and stent, the experimental approach only allowed PIV analysis of a limited region of the sagittal cross section.^47^ This area was identified in the numerical cross section, as indicated by the areas delimited by the white dashed border line in Fig. 4.

**Figure 5:**
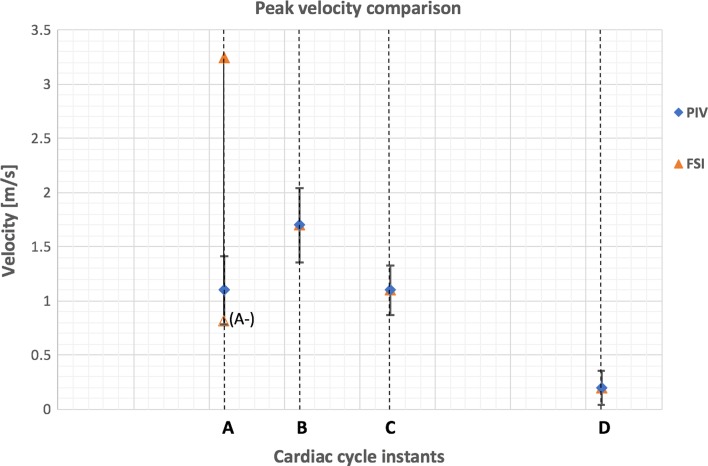
Comparison of the peak axial velocity between the two models. The standard deviation of the PIV data is displayed as the error bar.

A quantitative comparison based on the peak component of the velocity across the analysed region at the same instants of the cardiac cycle between the two models is presented in Fig. 5.

At instant ‘A’, i.e. when the flowrate reaches its maximum increase at the beginning of the valve opening, the main flow features of the FSI analysis are characterised by a diverging flow, supporting the opening of the leaflets, and a narrow centeralised fast jet flow with peak velocity of 3.2 m/s, compared with a broader jet and a peak velocity magnitude of 1.1 ± 0.31 m/s for the PIV study in the equivalent region. Due to the discretisation of the numerical results, the exact time equivalent of instant ‘A’ from the *in vitro* study falls between two timesteps from the computational simulation. The peak velocity identified as ‘A-’ in Fig. 5 indicates the peak velocity from the earlier of these two time instants, whilst the faster peak velocity marked in the chart represents the latter of these timesteps.

At peak systole (instant ‘B’ in Fig. 4), the valve cusps expand into the sinuses, the central jet deflects towards the sinus side of the aortic wall, and two slow flow recirculation zones develop at the proximal and distal outflow side of the leaflet, in both the numerical and *in vitro* models. A recirculation also forms above the commissure, maintaining this location throughout systole. A maximum ejection velocity of 1.7 m/s measured inside the *vena contracta* (the minimum diameter of the fast central jet) of the numerical model matches the value of 1.7 ± 0.34 m/s taken from the *in vitro* model (Fig. 5). At a smaller scale, the distribution of fluid velocity in the sinus is also similar between the two models, with a region of relatively high flow at the top of the sinus, adjacent to the root wall.

When the flow undergoes maximum deceleration (instant ‘C’ in Fig. 4), the vortical structures formed in the sinuses are still present in both models, whilst the jet flow, with peak velocity of 1.1 m/s in the computational analysis correlating with 1.1 ± 0.23 m/s in the experimental study, and the jet flow still angles towards the sinus side root wall. A comparable width of slow and return flow is evident on the commissure side of the root, with a similar diameter central jet flow across both investigations. The sinus flow distribution is again similar in the overlapping velocity fields of the two analyses, with the faster sinus flow concentrated in the upper region alongside the root wall.

At the end of systole (instant ‘D’ in Fig. 4), the two recirculations previously observed in the Valsalva sinus and above the commissure move towards the axis of the aorta in both the *in vitro* and *in silico* analyses. Again, the peak axial velocity of the numerical model, 0.2 m/s, matched the equivalent data from the *in vitro* investigation, 0.2 ± 0.16 m/s (Fig. 5).

**Figure 6:**
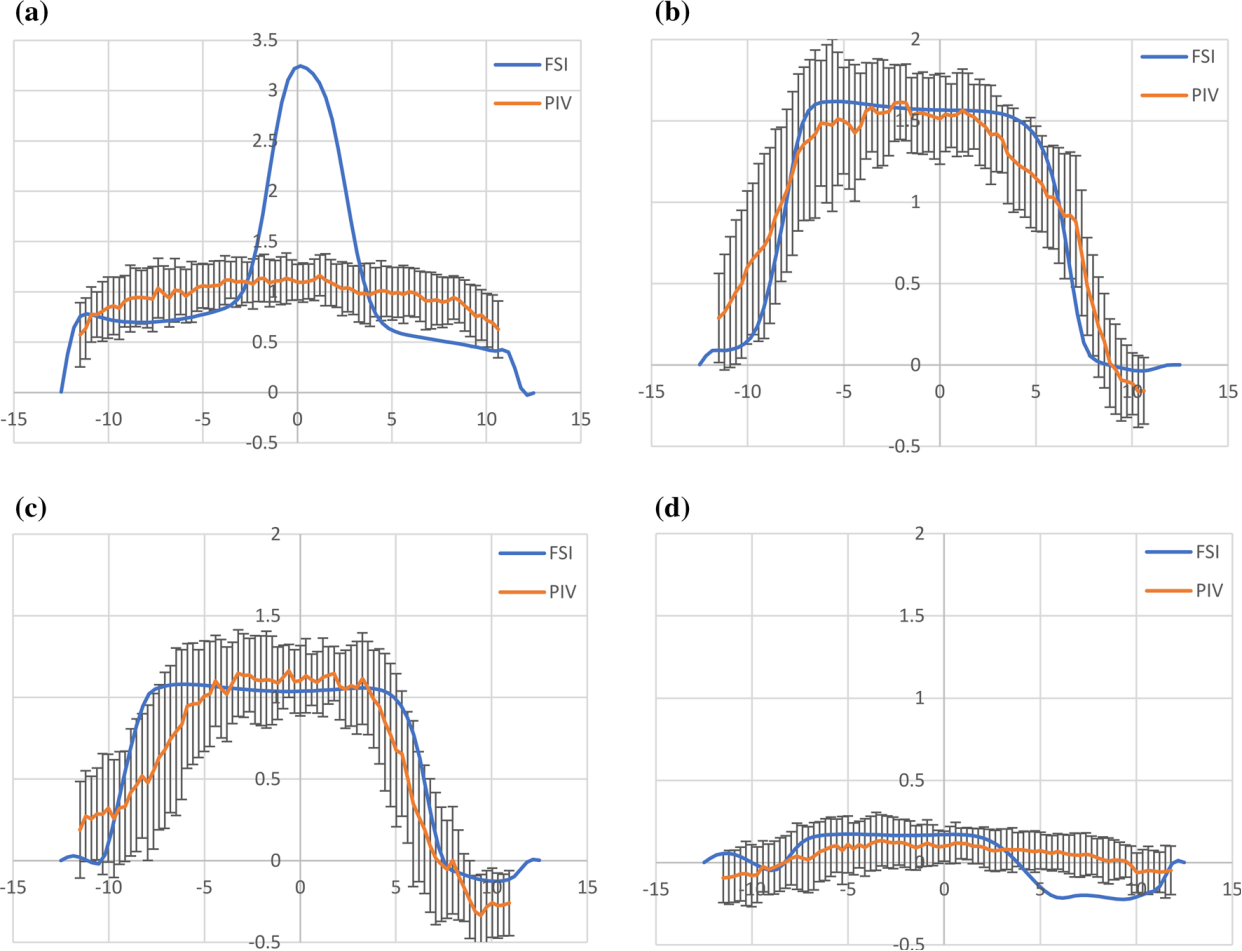
Comparison of the velocity profiles over the cross-section of the root at the Sino-Tubular junction and analysed at instants (a), (b), (c) and (d) of the cardiac cycle. The Particle Image Velocimetry (PIV) data includes an error bar representing the standard deviation of the measurements over 100 PIV image pairs.

Further quantitative comparison was carried out by correlating the velocity profiles of the *in vitro* and *in silico* studies at the sinotubular junction, as shown in Fig. 6.

Apart from the central jet portion of instant ‘A’, where the velocity ranges from 1.1 ± 0.15 m/s in the PIV analysis in contrast to a peak velocity of 3.2 m/s in the FSI analysis, the velocity profiles acquired from the numerical analysis were consistent with those from the *in vitro* data. For instants ‘B’–‘D’, 90% of the velocity magnitudes across the STJ of the numerical model were within the standard deviation of the velocity measurement for the *in vitro* model, with 96% matching for ‘B’, 96% for ‘C’, and 77% for ‘D’.

The EOA for the numerical simulation was calculated as 2.46 cm^2^, close to the value estimated from the *in vitro* investigation of 2.43 ± 0.02 cm^2^.^47^

### Healthy Physiological Mechanism

**Figure 7:**
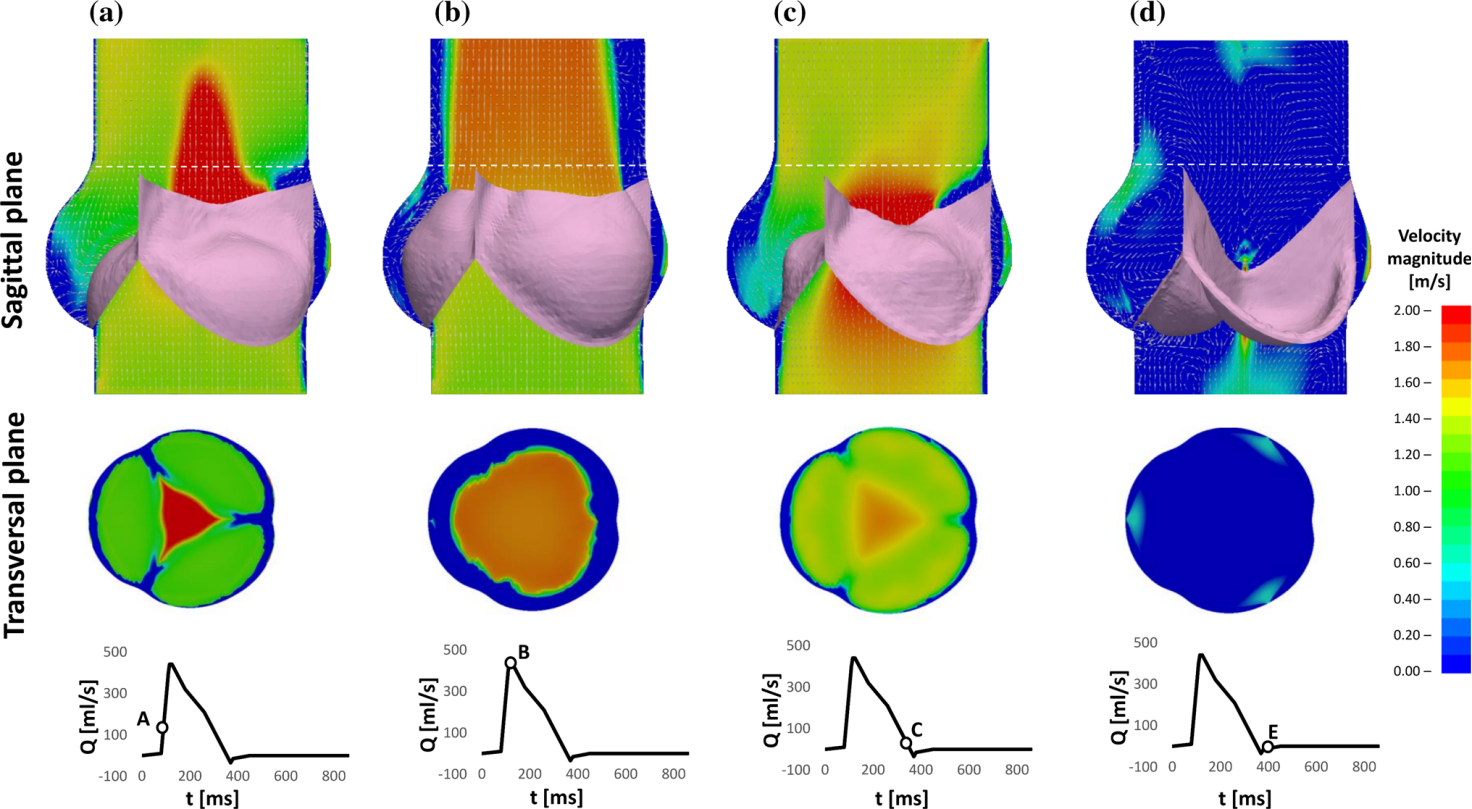
Velocity maps within the sinus and across the valve annulus at different instants of the cardiac cycle.

Velocity fields across a sagittal cross section (as in the previous validation) and a transversal cross section at the valve annulus obtained at the closest available time-step to the selected instants of the cardiac cycle used in the validation are shown in Fig. 7.

Analysis of the flow velocity maps and vectors indicates some major differences from the previous analysis. Opening is promoted by a radially expanding component of the flow, which develops in the early systolic phase, occupying most of the Valsalva sinuses region (instant ‘A’ in Fig. 7). Although the valve leaflets are unchanged in terms of geometry, boundary constraints and material properties, in the absence of the stent they undergo a much wider opening, taking a bulging shape closely matching the profile of the sinuses. As a result, the gap that forms between the leaflet and the aortic wall is reduced and does not allow the formation of large flow recirculations.

**Figure 8:**
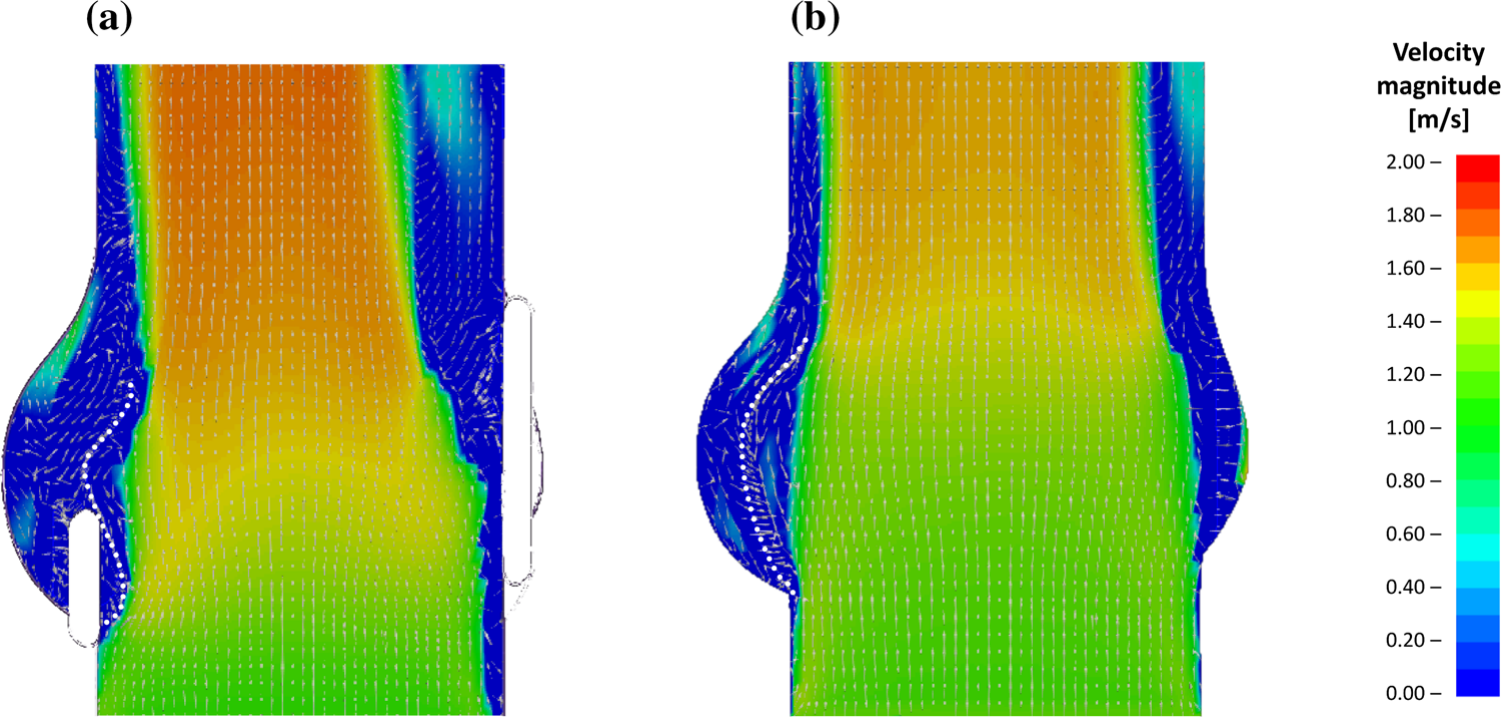
Velocity maps at the maximum leaflets opening obtained for the validation and for the physiological models.

A comparison of the sagittal cross section at the maximum opening of the leaflets for both the preliminary and physiologically adapted models is shown in Fig. 8.

**Figure 9:**
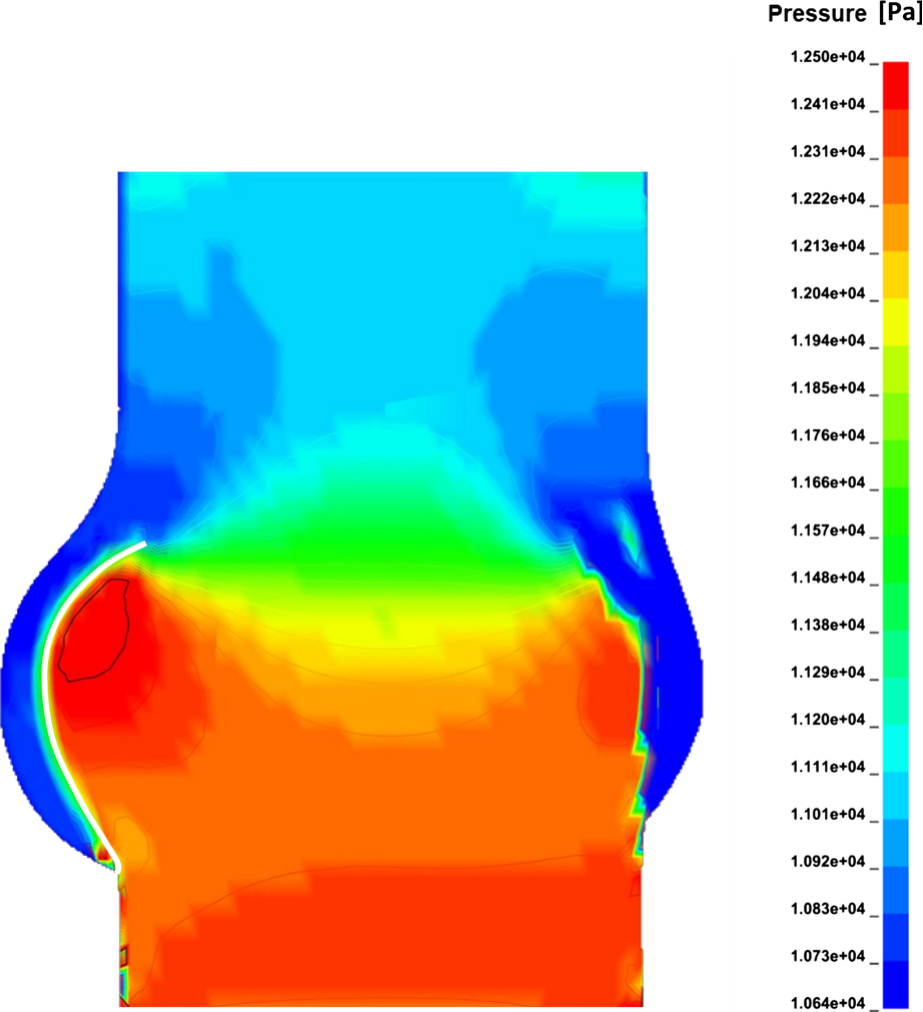
Pressure distribution at the systolic peak.

The comparison clearly shows how the presence of the stent determines a marked reduction in the ability of the leaflets to expand, leaving a much larger chamber between the valve and the sinus wall, thus enabling the formation of vortices within this volume. The physiological configuration promotes a mechanism which was not observed in the preliminary model, nor in the literature: as the central jet increases in velocity, the flow contraction at the leaflets’ exit generates a reduction in pressure which results in some suction in the gap between the leaflets and the sinus wall. This produces a trans-leaflet pressure difference, measured at around 15 mmHg in this simulation, which contributes to further expand the leaflets towards the sinuses wall. This process, which has a stronger effect when the ejected flow becomes faster, increases the EOA of the valve (from 2.46 cm^2^ in the preliminary model to 2.76 cm^2^ in the adapted model, an increase of 11%), improving the hydrodynamic valve performance. Figure 9 illustrates the map of the pressure distribution obtained at the timestep associated with the maximum flow velocity.

At the end of systole, as shown in Fig. 7d, the Valsalva sinuses play a primary role in promoting a centripetal flow which supports a prompt valve closing. Vortices become evident during the diastolic phase, when two large recirculations, one on top of the other, establish into the sinus, decreasing blood stagnation.

## Discussion

This study was undertaken to develop a reliable FSI model which provided a more accurate description of the haemodynamics expected in an idealized healthy physiological aortic root.

The first part of the study attempted to achieve a validation of the numerical approach by comparing the relevant features with corresponding PIV experimental measurements. Although 2D PIV has previously been used for the validation of computational studies of mechanical heart valves’ behavior,^10^,^22^ the reliability of FSI models of biological valves has generally been established by comparison of the valve opening and closing time with *in vivo* measurements.^31^,^34^,^36^ Where suitable *in vitro* experimental data have been available, the validity of the numerical analyses was supported by visual comparisons of the leaflets’ position or angular displacement throughout the cardiac cycle.^9^,^14^,^28^,^42^

In this study a more comprehensive validation, based both on quantifiable haemodynamic parameters and PIV findings, was performed to ascertain the reliability of the numerical results obtained with the preliminary model. This was fulfilled by analyzing the velocity maps obtained across a section of the aortic root, comparing the peak axial velocity and the velocity profiles at the STJ for 4 different instants in the systolic cycle, along with qualitative evaluation of the flow patterns at these instants. EOA was also acquired as a global hydrodynamic valve performance parameter for both techniques, enabling further quantitative comparison.

Achieving a satisfactory validation was not trivial, and involved the tuning of numerical parameters, such as the mesh grid resolution of the fluid and structure, the number of coupling points within the fluid–structure coupling definition, and the setting of appropriate algorithms able to contain the excessive distortion of the elements.

The qualitative and quantitative comparisons indicate a generally good agreement, confirming the consistency and periodicity of the results. The results were also closely matched in terms of EOA. The largest discrepancies were obtained for the velocity fields at instant ‘A’, corresponding to the opening of the valve, where a significant mismatch could be observed, though limited to the central region of the flow. This can be attributed to the fast dynamics that characterizes this phase of the cycle, for which any slight difference in the instant analysed may result in significantly different configurations. As a consequence, the two investigations for instant ‘A’ do not necessarily analyse the same degree of valve opening in the two studies. The higher velocity central flow observed in the computational analysis is due to the development of an initial orifice at the center of the valve, which can be observed for the first time at this timestep. On the contrary, the velocity distribution from the *in vitro* result suggests that this stage has not been reached yet. Analyzing the timestep before that presented as instant ‘A’, indicated by the datum designated as ‘A-’ in Fig. 5, resulted in a velocity distribution with no central jet, and lower magnitude than that of the experimental study, confirming that the numerical model was, in fact, reproducing similar flow dynamics to the *in vitro* model, but the timestep did not enable the display of the same exact instant from the experimental investigation. Differences may also result from the phase averaging over 100 cycles in the PIV study,^47^ wherein variations between cycles (e.g. different extents of valve opening) could lead to a broader and less intense central jet than in the numerical study.

In summary, the preliminary FSI model showed the ability to reproduce and capture the haemodynamic features detected in the experimental investigation, especially once valve opening has been completed.

This validated numerical framework was then altered by removing the presence of the stent and adjusting the physical properties of the fluid and vessel wall, to better represent the healthy native aortic valve. These changes resulted in major changes in the valve opening and functional mechanisms. In particular, during systole, the leaflets protrude much deeper into the Valsalva sinuses, reducing their propensity to generate and host the recirculation areas observed in both the experimental and preliminary numerical model. A key function of the sinuses appears to be that of providing a chamber able to host the cusps during systole, reducing the cusps’ impact on the flow through the valve. During ventricular ejection, the native aortic valve cusps expand very close to the vessel walls, enhancing the valve’s geometric orifice area. This effect is amplified by a suction that establishes in the gap between the leaflets and the aortic wall, due to a Venturi effect induced by the fast jet flow, which is strongest at the maximum flowrate. Vortical zones, which appear to be negligible during the vast majority of the systolic cycle, become significant during diastole, contributing to prevent blood stagnation.

This study suggests that presence of systolic vortices in the sinuses, subject of much of the literature to date,^4^,^40^,^47^ is not associated with healthy physiological operating conditions, but rather with stenotic dynamics due to calcifications, geometric mismatch or other non-physiological causes, such as the constricting presence of a supporting stent, as in the presented *in vitro* case.

## Conclusions

The use of numerical FSI models, validated with *in vitro* findings, has led to a more complete understanding of the physiological mechanisms that determine the aortic valve function. The study has shown an alternative phenomenon to what is currently described in the literature, where the function of vortical flow regions during the systolic phase appears strongly debunked.

The presented model can serve as a benchmark for the flow conditions associated with a healthy functioning mechanism of the aortic valve, providing enhanced valve performance indicators. This can represent an important basis to improve investigation of the haemodynamic changes produced in diseased and treated conditions, supplying a powerful tool in the design of novel and/or improved devices and therapies.

The study confirms the role that numerical approaches can play in the prediction of pathologies induced by flow alterations, providing a full view of the flow dynamics within the aortic root that are limited using experimental techniques.

## Limitations

Although the validated FSI model was shown to realistically and accurately simulate the fluid dynamics established in the aortic root, some assumptions still need to be considered. The shape and dimensions of the aortic valve and root are based on an idealized model assuming that the 3 Valsalva sinuses and their corresponding leaflets are identical, thereby introducing a 120-degree geometrical symmetry. In reality, the native aortic valve and root are characterised by individually specific shapes and dimensions. The native aorta is longer and more complex than that presented in this study, which could induce alterations to the fluid dynamics. Moreover, the presence of coronary arteries was not taken into account, and requires future studies, as does the effect of different degrees of wall compliance upon the haemodynamics of the region. Finally, the outlet of the fluid domain is relatively close to the aortic root so that the associated boundary layer effect cannot be fully eliminated.

## Electronic supplementary material

Below is the link to the electronic supplementary material.

Electronic supplementary material 1 (DOCX 4733 kb)
