# Supplementary material for: Validation and Extension of a Fluid–Structure Interaction Model of the Healthy Aortic Valve
Source: Cardiovasc Eng Technol. 2018 Nov 7;9(4):739–51. doi: 10.1007/s13239-018-00391-1 (PMC6290709; doi:10.1007/s13239-018-00391-1)
Supplement: Supplementary file 1 — Electronic supplementary material 1 (DOCX 4733 kb) [file 13239_2018_391_MOESM1_ESM.docx]

**MESH GRID INDEPENDENCE**

Element dimensions were chosen after performing a mesh grid independence analysis on three different fluid and structure meshes: coarse (approximately 15.000 elements for the fluid and 10.000 for the shell), medium (approximately 110.000 elements for the fluid and 25.000 for the shell), and fine (approximately 900.000 elements for the fluid and 100.000 for the shell).

The results of the related FSI analyses were compared in terms of velocity profiles extracted along the vertical axis from the annulus height to the STJ height, as indicated by the dashed line in Figures A1, A2, A3 and A4, which represented the area that most influenced the valvular dynamics.

The analysed instants of the cardiac cycle correspond to the same instants used for the model validation but, in this case, taken during the first cycle. This was due to the exceedingly expensive computational time required to run the numerical analysis with the fine mesh grid.

The velocity maps obtained at a cross section of the aortic root at the specific instants for the three different mesh models are reported in Figures A1-A4. The region where the maximum velocity magnitude was observed is contoured by a red square.


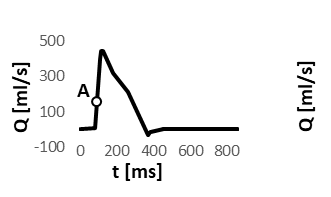


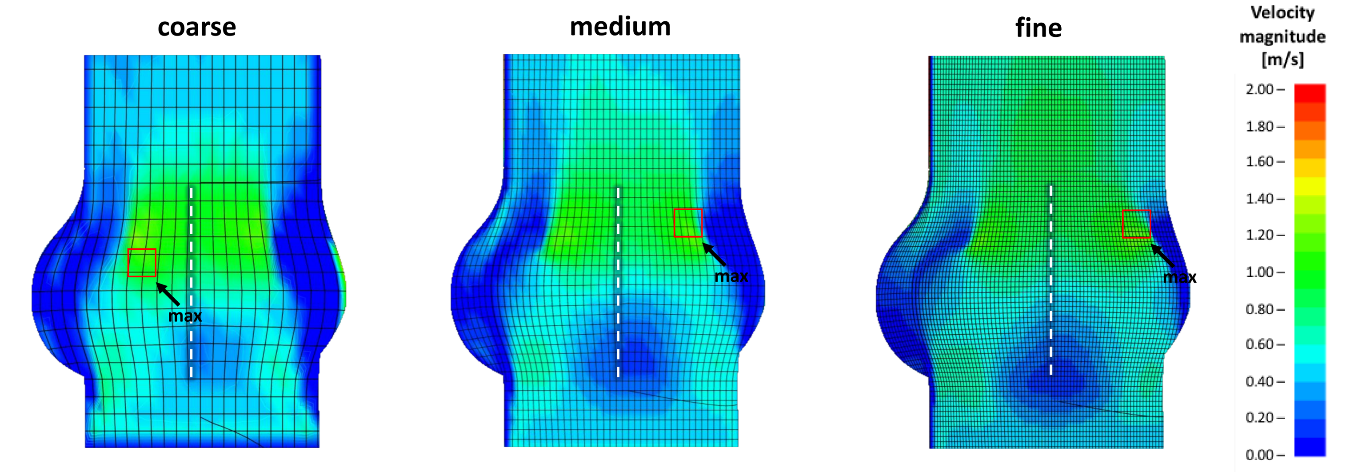


Figure A1. Velocity maps at instant A


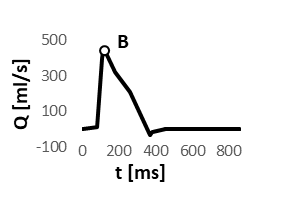


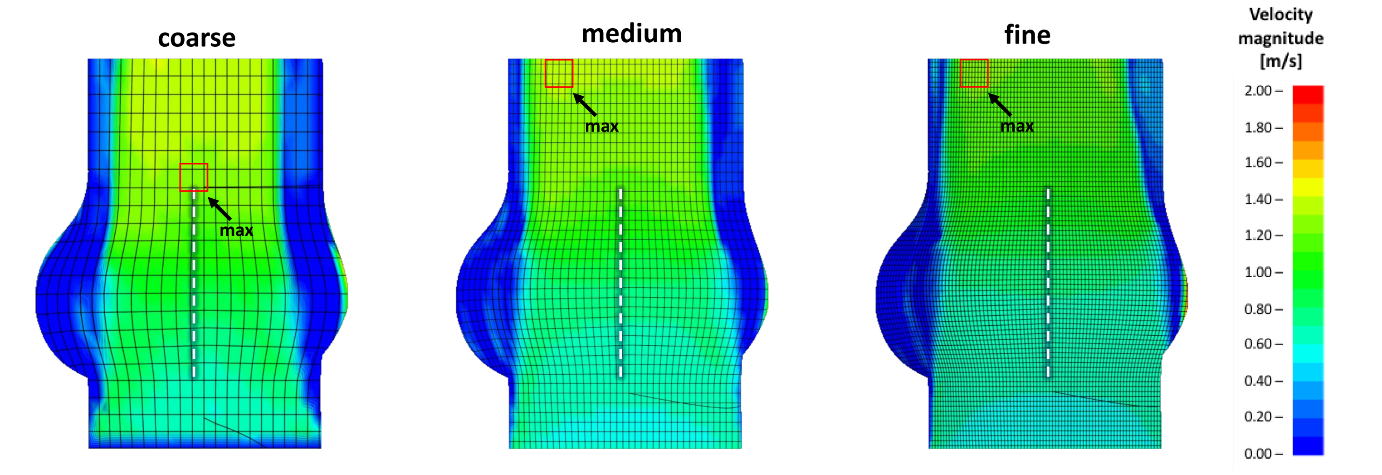


Figure A2. Velocity maps comparison at instant B.

**
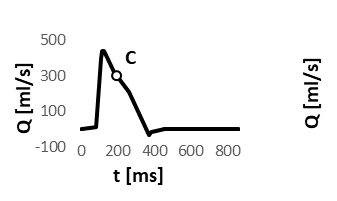
**

**
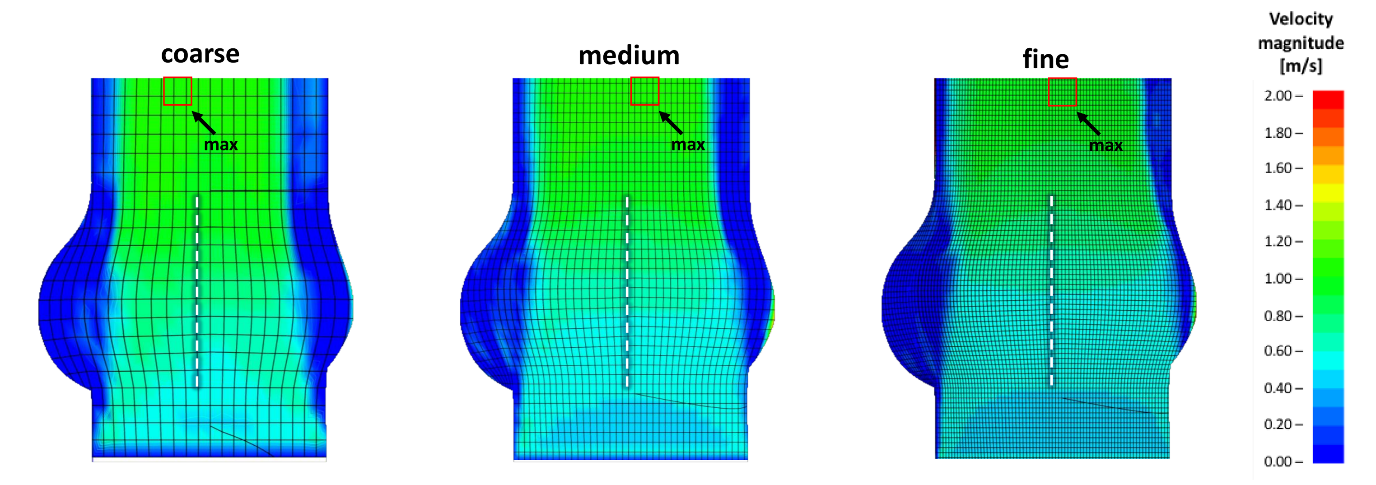
**

Figure A3. Velocity maps comparison at instant C.


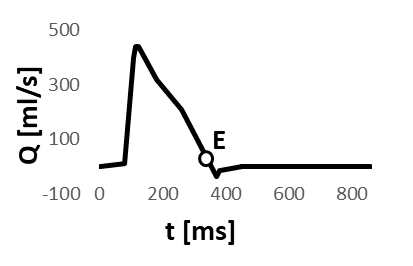


**
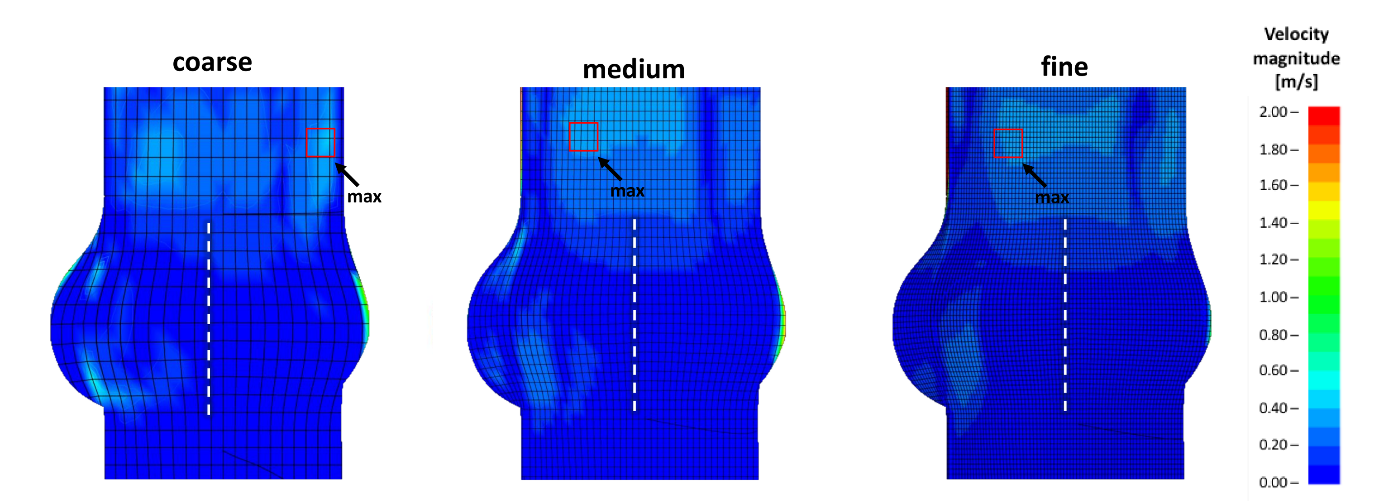
**

Figure A4. Velocity maps comparison at instant D.

From the comparison of the velocity profiles along the axis, the average discrepancy was found to vary from 0.03 m/s and 0.09 m/s between the medium and coarse mesh grid, and from 0.007 m/s to 0.08 m/s between the medium and fine mesh grid during the analysed instants of the cardiac cycle. In both cases, the average variations were less than two order of magnitudes lower than the peak systolic velocities. However, the valve in the coarse model was not able to close properly, leading to an unphysiological behavior during diastole.

The location where the maximum velocity was observed is consistent for the medium and fine meshes, confirming similar distributions, while regularly displaced for the coarse mesh.

The results were also compared in terms of maximum in plane stress experienced by the leaflets. As an example, Figure A5 shows distribution of the maximum in plane stress obtained at the peak of systole for the three meshes. Comparison of the stress levels for the entire cycle showed a variation in peak values between the medium and the fine mesh within 10% at the valve opening, and within 5% at the valve closure, resulting in acceptable level of grid convergence. Variation percentages of 34% and 11% were observed between the medium and coarse mesh grid at the valve opening and closure, respectively.


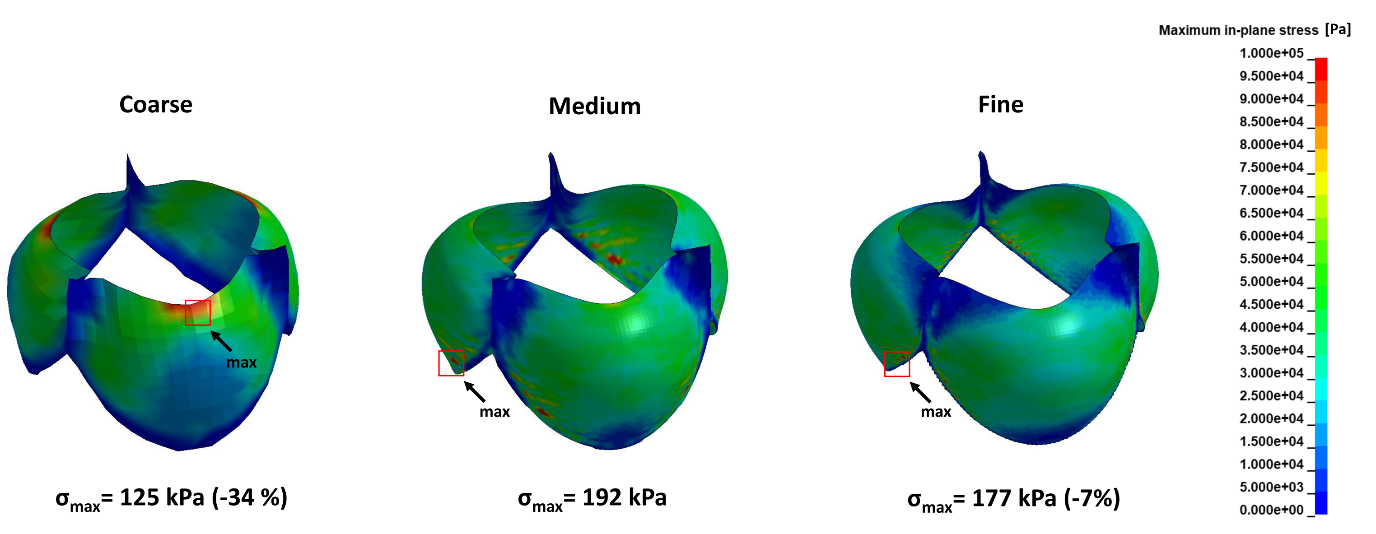


Figure A5. Comparison of the maximum in plane stress at the valve opening.

Hence, since the medium size mesh grid achieved an acceptable level of grid convergence, both in terms of fluid and structural parameters, this was selected to perform the study.
